# Prognostic Impact of Body Mass Index in Atrial Fibrillation

**DOI:** 10.3390/jcm13113294

**Published:** 2024-06-03

**Authors:** Maria Nteli, Despoina Nteli, Dimitrios V. Moysidis, Anastasia Foka, Panagiotis Zymaris, Triantafyllia Grantza, Olga Kazarli, Alexis Vagianos, Andreas S. Papazoglou, Anastasios Kartas, Athanasios Samaras, Alexandra Bekiaridou, Efstathios Spyridonidis, Antonios Ziakas, Apostolos Tzikas, George Giannakoulas

**Affiliations:** 1 First Department of Cardiology, AHEPA University Hospital, School of Medicine, Faculty of Health Sciences, Aristotle University of Thessaloniki, 54636 Thessaloniki, Greece; marintel@gapps.auth.gr (M.N.); despntel@gapps.auth.gr (D.N.); anastf00@gmail.com (A.F.); zymarisp@gmail.com (P.Z.); filiagrantza@gmail.com (T.G.); olgakaz14@gmail.com (O.K.); alexvagianos@gmail.com (A.V.); tkartas@gmail.com (A.K.); ath.samaras.as@gmail.com (A.S.); ampekiaridou@gmail.com (A.B.); tonyziakas@hotmail.com (A.Z.); 2 424 General Military Hospital of Thessaloniki, 56429 Thessaloniki, Greece; dimoysidis@gmail.com (D.V.M.); stathisspyridonidis@gmail.com (E.S.); 3 Athens Naval Hospital, 11521 Athens, Greece; anpapazoglou@yahoo.com; 4 Feinstein Institutes for Medical Research, Northwell Health, Manhasset, NY 11030, USA; 5 Interbalkan European Medical Center, 55535 Thessaloniki, Greece; aptzikas@yahoo.com

**Keywords:** atrial fibrillation, body mass index, major cardiovascular events, obesity paradox

## Abstract

**Background/Objectives**: Contradictory results have been reported regarding the influence of obesity on the prognosis of atrial fibrillation (AF). The present study aimed to explore the potential association of body mass index (BMI) with the clinical outcomes of hospitalized patients with AF. **Methods**: In this retrospective, post hoc analysis of the MISOAC-AF randomized trial, 1113 AF patients were included and stratified as the following: underweight (BMI < 18 kg/m^2^), normal weight (BMI 18–24.9 kg/m^2^), overweight (BMI 25–29.9 kg/m^2^), and obese (BMI ≥ 30 kg/m^2^). The primary outcome was all-cause mortality; the secondary composite outcome was any hospitalization related to AF, heart failure (HF), or stroke. Cox regression analysis, survival analysis, and spline curve models were utilized. **Results**: Of the patients (median age: 76 years (IQR: 13), male: 54.6%), the majority were overweight (41.4%), followed by obese (33%), normal weight (24%), and underweight (1.6%). During a median 31-month follow-up, 436 (39.2%) patients died and 657 (59%) were hospitalized due to AF, HF, or stroke. Underweight, overweight, and obesity groups were significantly associated with an increased risk of all-cause mortality (*p*-values 0.02, 0.001, and <0.001, respectively), while overweight and obesity were significantly associated with the composite endpoint (*p*-values 0.01, <0.001, respectively) compared to normal weight. The spline curve analyses yielded that BMIs > 26.3 and > 25 were incrementally associated with all-cause mortality and the composite endpoint, respectively. A J-shaped relationship between BMI and AF prognosis was deduced. **Conclusions**: In conclusion, in recently hospitalized AF patients, BMI values outside the normal range were independently associated with poorer prognosis; therefore, it is essential that AF patients maintain a normal weight.

## 1. Introduction

Atrial fibrillation (AF) constitutes the most frequently observed cardiac arrhythmia [1]. AF is a marker of increased risk for stroke, heart failure (HF), ischemic heart disease, or chronic kidney disease [2,3,4,5]. Obesity, defined by a body mass index (BMI) equal to or greater than 30 kg/m^2^ [6], has gained epidemic proportions with dramatic consequences worldwide mainly due to lifestyle components such as sedentary behavior, physical inactivity, and dietary habits [7,8]. Many epidemiological studies point out obesity as an independent risk factor for developing AF as well as for increased disease burden [9,10,11,12,13,14,15]. Based on the results of some studies a 10% reduction in weight and a BMI < 27 kg/m^2^ has been recommended for AF patients to reduce disease burden [16,17]. Nonetheless, the effect of BMI on hard clinical endpoints in patients with AF remains unclear [18]. Several studies indicate that increased BMI might be associated with favorable clinical outcomes in AF and other cardiovascular diseases; a phenomenon known as the “Obesity Paradox” [19,20,21]; however, few publications exist, reaching a contradictory conclusion–namely indicating that overweight/obese individuals with AF do not present with a better prognosis [22,23,24]. Hence, the existing body of evidence on the clinical outcomes of AF in overweight or obese patients warrants further validation. Aiming to add to the existing literature, we investigated the association of obesity (and the whole BMI spectrum) with the risk of all-cause mortality and rehospitalization, using data from a cohort of well-characterized patients with AF.

## 2. Materials and Methods

### 2.1. Study Design

This is a retrospective observational study based on the MISOAC-AF (Motivational Interviewing to Support Oral AntiCoagulation adherence in patients with non-valvular Atrial Fibrillation) randomized clinical trial (ClinicalTrial.gov identifier: NCT02941978 accessed on 30 May 2024). The study took place at the cardiology clinic of a tertiary academic hospital located in Thessaloniki, Greece, and its design and main outcomes were detailed in prior publications [25,26]. In summary, MISOAC-AF evaluated the impact of patient–physician interviews and standardized guidance on the adherence of patients with AF to oral anticoagulation (OAC) therapy.

### 2.2. Data Sources/Study Population

Our analysis included data derived from patients with AF and available BMI values, who were discharged after hospitalization between December 2015 and June 2018. The MISOAC-AF electronic database contained baseline characteristics, medical history, concomitant medication, laboratory and echocardiographic data, discharge diagnoses, and vital status. These data were obtained from electronic hospital records and by physicians who interviewed the patients while they were hospitalized. Every study participant provided written informed consent. The study complied with the 1975 Declaration of Helsinki and received approval from the ethics committee of Aristotle University of Thessaloniki.

### 2.3. Definitions

AF was defined as an electrocardiographic arrhythmia newly diagnosed or previously documented. Specifically, AF had to be recorded as an irregular heart rhythm lasting more than 30 s, characterized by the absence of sinus P waves and varying R-R intervals. BMI values were derived from the equation weight/(height)^2^ (kg/m^2^) of the enrolled patients. Patients were classified according to their BMI at index hospitalization as follows:

(i) under 18: underweight;
(ii) from 18 to 24.9: normal weight;
(iii) from 25 to 29.9: overweight;
(iv) from 30 to 34.9: obese (obese class I);
(v) from 35 to 39.9: severely obese (obese class II);
(vi) equal to or over 40: morbidly obese (obese class III).

### 2.4. Study Outcomes

The primary outcome was all-cause mortality (death from any cause). Any hospitalization related to AF or HF (e.g., arrhythmia, decompensated HF, pacemaker/defibrillator implantation, hemorrhage, thromboembolism) or stroke constituted the secondary composite outcome. The follow-up process was carried out in the first, second, sixth, and twelfth post-discharge months and then annually until April 2020. The website of the National Insurance System of Greece was utilized for validation of the reported deaths. 

### 2.5. Statistical Analysis

The baseline characteristics of patients were analyzed in relation to BMI. Each continuous and categorical variable was described by mean–standard deviation (SD), median–interquartile range (IQR), and frequency–percentage, respectively. The comparison between continuous variables was conducted using one-way ANOVA or Kruskal–Wallis test, while Pearson’s Chi-squared test or Fisher’s exact test was performed for the comparison between categorical variables. A Kaplan–Meier survival analysis was implemented to examine the association of BMI with the primary and secondary composite outcomes. A log-rank test was utilized to compare the all-cause mortality and hospitalization in different BMI groups. Adjusted hazard ratios (aHR) were calculated through a multivariate Cox regression analysis in which the following—univariately significant or clinically important—variables were included for adjustment: age, gender, smoking, history of HF, history of coronary artery disease, hypertension, diabetes mellitus (DM), CHA_2_DS_2_-VASc score, glomerular filtration rate (GFR), left ventricular ejection fraction (LVEF) on transthoracic echocardiogram, and AF subtype (first diagnosed, paroxysmal/atrial flutter, persistent/permanent). The relationship between the continuum of BMI values and the risk of primary and secondary outcomes was illustrated via restricted cubic spline curves. *p*-values < 0.05 were considered statistically significant. SPSS software, version 26 (IBM SPSS Statistics, IBM Corporation, New York, USA) and R version 3.4.4 (R Foundation for Statistical Computing, Vienna, Austria) were employed for data management, statistical analysis, and graph creation.

## 3. Results

All of the 1113 patients with AF enrolled in the MISOAC-AF trial fulfilled the inclusion criteria for the present study. Of them, 608 (54.6%) were men. The median age of study participants was 76 years (IQR: 13), while the median BMI of study participants was 27.8 (IQR: 6.3). A total of 18 patients (1.6%) constituted the underweight class (BMI < 18), 267 patients (24%) the normal weight class (BMI 18–24.9), 461 patients (41.4%) the overweight class (BMI 25–29.9), 240 patients (21.6%) the obese class (BMI 30–34.9), 87 patients (7.8%) the severely obese class (BMI 35–39.9), and 40 patients (3.6%) the morbidly obese class (BMI ≥ 40). 

Baseline clinical, laboratory, and echocardiographic characteristics are described in Table 1 according to different BMI categories.

In general, underweight patients had no significant differences in comparison to normal-weight patients regarding their baseline characteristics. On the contrary, overweight, obese, severely obese, and morbidly obese patients differed in the prevalence of HF and DM along with the median age when compared to normal-weight patients (*p*-values < 0.05).

In total, 7 (38.9%) of the 18 underweight patients, 73 (27.3%) of the 267 normal-weight patients, 171 (37.1%) of the 461 overweight patients, 112 (46.7%) of the 240 obese patients, 49 (56.3%) of the 87 severely obese patients, and 24 (60%) of the 40 morbidly obese patients died during a median 2.6-year follow-up. With regards to the secondary outcomes, 11 (61.1%) underweight, 118 (44.2%) normal weight, 269 (58.4%) overweight, 158 (65.8%) obese, 68 (78.2%) severely obese, and 33 (82.5%) morbidly obese patients were hospitalized due to AF-, HF- or stroke-related events.

**Figure 1:**
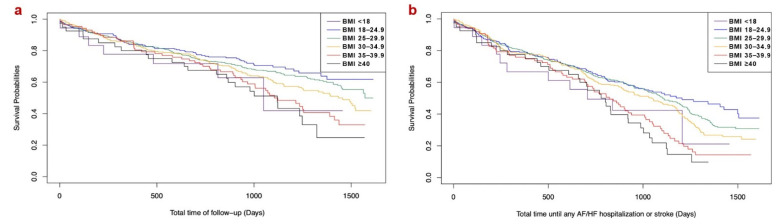
Kaplan–Meier analysis on (**a**) all-cause mortality and (**b**) any hospitalization related to AF, HF, or stroke (AF = atrial fibrillation, HF = heart failure, BMI = body mass index).

**Figure 2:**
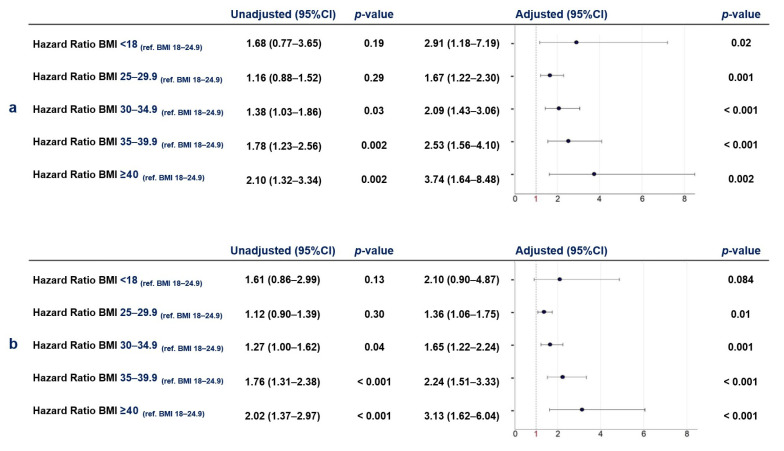
Risk of primary (**a**) and secondary (**b**) outcomes according to BMI category (BMI = body mass index, 95% CI = 95% confidence interval). The normal-weight BMI category (BMI = 18–24.9) was used as reference (ref.) for all comparisons.

The Kaplan–Meier curves for all-cause mortality and any hospitalization related to AF, HF, or stroke are shown in Figure 1 (log-rank test: both *p*-values < 0.001). Multivariate Cox regression analysis yielded that BMI values ≥ 25 were independently associated with higher risk for both the primary and the secondary outcomes (*p*-values < 0.05) compared to the normal-weight patients, while BMI values < 18 were significantly associated with higher risk for the primary outcome (*p*-value < 0.05) compared to the normal weight category (Figure 2).

**Figure 3:**
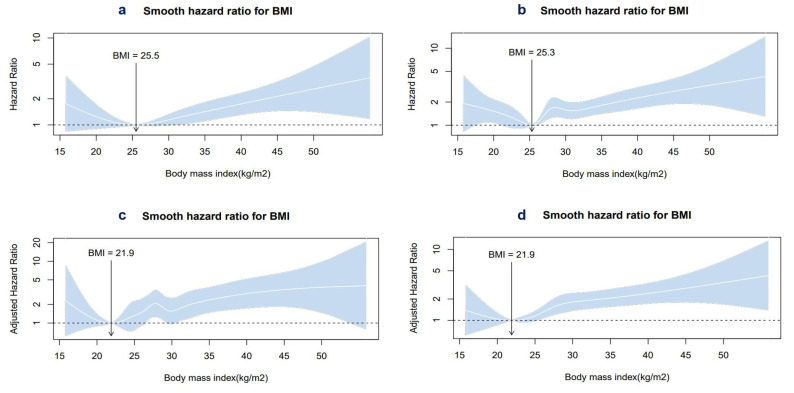
Cubic spline curve for the association between (**a**) BMI and all-cause death, (**b**) BMI and any hospitalization related to AF, HF, or stroke, (**c**) BMI and all-cause death after multivariate adjustment, and (**d**) BMI and any hospitalization related to AF, HF, or stroke after multivariate adjustment (BMI = body mass index, AF = atrial fibrillation, HF = heart failure).

The unadjusted and adjusted spline curves illustrating the risk of death as well as AF-, HF-related hospitalization, or stroke in patients with AF across the BMI spectrum are presented in Figure 3. BMI values from 26.3 to 29.7 and >30.7 positively correlated with the probability for death, while BMI values > 25 emerged as independent predictors of the composite clinical endpoint “any hospitalization related to AF, HF, or stroke”. BMI values < 21.9 were also linked to an enhanced risk of the studied outcomes, albeit not significantly. 

## 4. Discussion

In this real-world analysis, underweight, overweight, and obese patients had an independently increased risk of all-cause mortality and hospitalization related to AF, HF, or stroke when compared to normal-weight patients. 

Despite the association between obesity and the risk of AF incidence being already well-established [10,27,28], the exact relationship between BMI and prognosis in AF patients is yet to be fully elucidated, since the relevant studies have reached equivocal results.

Several studies indicate that, unanticipatedly, the medically unfavorable phenotype of BMI ≥ 25 (and especially overweight and obesity class I) conduces to lessened prevalence of all-cause or cardiovascular death and thromboembolism among AF subjects, an observation described in the literature as the “Obesity Paradox” [29,30,31,32]. Our findings are inconsistent with this theory. A couple of meta-analyses affirmed the reduced risk of all-cause death, stroke, and thromboembolism inflicted on overweight and obese AF patients [33,34]. Intriguingly, the observed trends were mainly driven from the secondary analysis of randomized clinical trial data, while observational studies, and especially those with a prolonged follow-up duration, reached more ambiguous conclusions or failed to ascertain the “Obesity Paradox” [32]. 

Nevertheless, there exists a portion of the bibliography suggesting that BMI does not mediate the AF prognosis [18,35,36] and other researchers yielding results similar to ours, namely demonstrating that elevated BMI is linked to worse prognosis [22,23,24,37]. Specifically, in the latter studies, overweight and obese AF patients seemed to encounter increased thromboembolic risk (a 1 kg/m^2^ BMI elevation was equivalent to a 9% enhancement of the thromboembolism rates) and even reduced survival rates compared to their thinner counterparts.

There are ongoing discussions over whether the “Obesity Paradox” reflects a factual phenomenon [21,38], although this concept stands true for other heart-related diseases (e.g., hypertension, coronary artery disease, HF) alongside non-cardiovascular maladies (e.g., end-stage renal disease, DM) [21]. Multiple underlying mechanisms have been hypothesized in an attempt to decipher this conundrum. Problems inherent to the methodology utilized by some researchers, such as selection bias, could influence the reliability of the extracted results [39,40]. Moreover, within the overweight/obese groups, common cardiovascular diseases (such as hypertension and dyslipidemia)—including AF itself—may be identified earlier since obesity is a known risk factor for them; therefore, these patients usually receive earlier, more aggressive treatment [39]. Meanwhile, individuals categorized as having a normal or decreased BMI may not be truly healthy but suffer from some illness that leads to catabolism or to a pro-inflammatory state [18,40]. Lastly, there is increasing evidence that the most decisive role when it comes to AF prognosis might be played by cardiorespiratory fitness and not BMI itself [40]. Even in studies confirming the “Obesity Paradox”, it is stated that cardiorespiratory fitness would mitigate—if not neutralize—the paradox, resulting in the so-called “fat but fit” phenomenon [39,40]. 

The other conclusion of our research, namely that underweight AF patients are at increased risk for mortality and thromboembolism, is in alignment with the preponderance of the existing literature, including recent meta-analyses [32,33,41,42]. Possible pathophysiological mechanisms for this observed “Lean Paradox” include increased frailty and vulnerability to illness due to malnutrition or cachexia; enhanced activation of the renin–angiotensin–aldosterone system in response to stress; worse endothelial function combined with attenuated systemic inflammation; and increased levels of adipokines [33]. It should also be considered that underweight AF patients tend to be older and suffer from other chronic comorbidities (such as cancer) [32]. 

In light of the aforementioned reasoning, purposeful weight loss seems to be of pivotal importance in patients with established AF (apart from the ones with concomitant HF [43,44,45])—as proposed in the ESC recommendations for AF management [46]—due to alleviating the severity of AF symptomatology and improving recurrence-free survival [47,48]. A multidisciplinary approach should be employed in relation to AF management, incorporating its three traditional pillars (anticoagulation, rhythm control, and rate control) along with a comprehensive risk factor control, which can—among others—be achieved through ensuring that individuals maintain a normal BMI/percentage of lean body mass and fitness level [46,49,50]. However, further research should be conducted to clarify whether dietary interventions indeed yield improvements in long-term hard clinical AF endpoints [38,40] and untangle the conflicting landscape regarding the efficacy of pharmaceutical (such as anorexigenic drugs) and surgical (such as bariatric surgery) methods aiming at weight loss [38]. 

### Limitations

Certain factors place constraints on the generalizability of this study’s conclusions. The small size of our sample (especially regarding the underweight category) may lead to insufficiently powered results and type II errors. Additionally, this subgroup analysis was not prespecified in the MISOAC-AF protocol, thereby suffering from limitations associated with such a retrospective, non-randomized, single-center study. Moreover, in this study, only acutely hospitalized patients were included and most of these patients presented with poor cardiovascular risk factor control on admission. Consequently, our findings might not be reproducible on larger or non-hospitalized populations with AF. BMI, though predominantly utilized in clinical practice, is unable to discriminate between adiposity and lean mass. Alternative measures, such as waist circumference, waist-to-hip ratio, bioimpedance, 3D scanning, and dual-energy X-ray absorptiometry could be implemented as complementary indicators in further investigation. Since BMI fluctuation might influence rhythm control, another potential limitation is that BMI was measured only once (at baseline). Finally, despite the utilization of multivariate adjustment, these results are still vulnerable to unrecognized residual confounding. For instance, it has been previously discussed concerning underweight patients with AF that it is possible that their comorbidities, such as sarcopenia, could play a significant role in their prognosis, rather than the arrhythmia per se. Likewise, an overweight or obese status is associated with increased rates of comorbidities, such as type 2 DM, respiratory disorders, and chronic kidney disease, which are predictors of worse prognosis. This could imply that our findings may be influenced by obesity-related outcomes.

## 5. Conclusions

In conclusion, the present study demonstrated a higher mortality risk in both overweight/obese and underweight patients, which formed a J-shaped spline curve across the BMI spectrum. Therefore, AF patients should maintain their weight within the normal range. We hope our findings stimulate further investigation of the subject that will allow for more reliable risk assessment and, in practical terms, more effective risk management.

## Figures and Tables

**Table 1:** Baseline characteristics stratified by BMI category.

Total: 1113	Underweight	Normal Weight	Overweight	Obese	Severely Obese	Morbidly Obese	*p*-Value
Number of Cases	18 (1.6)	267 (24.0)	461 (41.4)	240 (21.6)	87 (7.8)	40 (3.6)	
Demographics							
Age (years)	74.6 (10.2)	78 (12.0)	76 (14.0)	75 (15.0)	72 (16.0)	68.8 (10.5)	<0.001
Female Gender	8 (44.4)	119 (44.6)	187 (40.6)	115 (47.9)	50 (57.5)	26 (65.0)	0.006
Cardiovascular Risk Factors and Comorbidities							
Smoking	10 (66.7)	159 (61.6)	276 (61.1)	134 (58.0)	47 (57.3)	24 (61.5)	0.925
Hypertension	14 (87.5)	205 (78.2)	374 (82.0)	191 (82.7)	70 (82.4)	37 (92.5)	0.344
Dyslipidemia	4 (26.7)	99 (37.9)	227 (49.9)	132 (57.1)	44 (53.7)	21 (52.5)	<0.001
Diabetes Mellitus	2 (12.5)	83 (31.4)	133 (29.3)	88 (37.9)	46 (54.8)	18 (45.0)	<0.001
HF including new cases at discharge HFrEF HFmrEF HFpEF	9 (52.9) 4 (23.5) 3 (17.6) 2 (11.8)	144 (56.5) 55 (21.6) 29 (11.4) 60 (23.5)	221 (48.9) 77 (17.0) 46 (10.2) 98 (21.7)	110 (47.6) 28 (12.1) 23 (10.0) 59 (25.5)	52 (61.2) 10 (11.8) 7 (8.2) 35 (41.2)	28 (73.7) 4 (10.5) 7 (18.4) 17 (44.7)	0.008 0.050 0.474 <0.001
AMI during or prior to hospitalization	5 (33.3)	48 (18.5)	103 (22.7)	45 (19.5)	21 (25.3)	7 (17.5)	0.436
CAD	9 (52.9)	95 (37.8)	194 (44.9)	98 (42.8)	33 (40.7)	10 (26.3)	0.153
Types of AF First-Diagnosed Paroxysmal Persistent Permanent	1 (7.7) 5 (38.5) 0 (0.0) 7 (53.8)	22 (8.5) 82 (31.8) 18 (7.0) 136 (52.7)	68 (15.2) 158 (35.3) 29 (6.5) 192 (43.0)	24 (10.7) 72 (32.0) 13 (5.8) 116 (51.6)	13 (15.9) 24 (29.3) 3 (3.7) 42 (51.2)	6 (15.4) 8 (20.5) 1 (2.6) 24 (61.5)	0.102 0.436 0.857 0.047
Cardiac Arrest	1 (7.1)	10 (3.9)	11 (2.4)	5 (2.2)	4 (4.9)	0 (0.0)	0.340
Prior Ablation	0 (0.0)	8 (3.1)	7 (1.5)	3 (1.3)	2 (2.4)	1 (2.6)	0.577
History of PCI/CABG	5 (29.4)	75 (28.8)	154 (33.8)	72 (31.0)	24 (28.2)	8 (20.0)	0.432
Pacemaker	0 (0.0)	18 (6.9)	28 (6.2)	12 (5.2)	5 (6.0)	0 (0.0)	0.613
ICD	0 (0.0)	15 (5.7)	7 (1.5)	8 (3.4)	6 (7.2)	0 (0.0)	0.012
Prosthetic Valve	0 (0.0)	24 (9.2)	25 (5.5)	7 (3.0)	3 (3.6)	1 (2.5)	0.068
Vascular Disease	5 (35.7)	116 (44.8)	217 (48.0)	105 (45.5)	48 (57.8)	15 (37.5)	0.222
Congenital Heart Disease	0 (0.0)	9 (3.4)	10 (2.2)	4 (1.7)	1 (1.2)	1 (2.5)	0.789
Thyroid Disease	4 (28.6)	59 (22.8)	92 (20.3)	44 (19.0)	20 (24.4)	14 (35.0)	0.244
COPD	5 (33.3)	36 (13.8)	48 (10.6)	33 (14.3)	12 (14.5)	10 (25.0)	0.024
Pulmonary Disease	5 (33.3)	40 (15.3)	55 (12.1)	34 (14.7)	16 (19.3)	16 (40.0)	<0.001
CKD	3 (21.4)	37 (14.2)	67 (14.8)	31 (13.4)	18 (22.0)	9 (23.1)	0.263
History of Stroke/TIA/Unspecified Stroke/Systemic Thromboembolism	3 (17.6)	52 (19.9)	80 (17.5)	48 (20.6)	16 (18.6)	2 (5.0)	0.226
History of Hemorrhagic Stroke	0 (0.0)	2 (0.8)	5 (1.1)	0 (0.0)	0 (0.0)	0 (0.0)	0.640
History of Major Bleeding	3 (18.8)	44 (16.9)	74 (16.2)	23 (9.8)	8 (9.4)	4 (10.0)	0.089
Bleeding while under OAC	9 (56.3)	81 (31.5)	131 (29.3)	56 (24.5)	23 (28.0)	13 (32.5)	0.116
Medication							
Antiplatelets on admission Aspirin Clopidogrel Both	4 (28.6) 2 (14.3) 1 (7.1) 1 (7.1)	58 (23.4) 26 (10.4) 22 (8.8) 10 (4.0)	131 (29.4) 69 (15.5) 33 (7.4) 29 (6.5)	71 (31.1) 35 (15.4) 22 (9.6) 14 (6.1)	17 (21.8) 9 (11.5) 5 (6.4) 3 (3.8)	4 (10.8) 1 (2.7) 3 (8.1) 0 (0.0)	0.044 0.123 0.909 0.425
VKA on admission	4 (28.6)	89 (35.7)	126 (28.3)	55 (24.0)	26 (33.3)	12 (32.4)	0.105
NOAC on admission Dabigatran Rivaroxaban Abixaban	3 (21.4) 0 (0.0) 2 (14.3) 1 (7.1)	81 (32.5) 13 (5.2) 38 (15.3) 30 (12.0)	139 (31.2) 36 (8.1) 52 (11.7) 51 (11.5)	87 (38.0) 22 (9.6) 41 (17.9) 24 (10.5)	31 (39.7) 8 (10.3) 15 (19.2) 8 (10.3)	16 (43.2) 1 (2.7) 8 (21.6) 7 (18.9)	0.229 0.295 0.131 0.772
Rate Control Medication on admission Beta-blockers Digoxin Both	12 (85.7) 11 (78.6) 0 (0.0) 1 (7.1)	177 (71.1) 138 (55.4) 13 (5.2) 26 (10.4)	301 (67.6) 270 (60.7) 13 (2.9) 18 (4.0)	167 (72.9) 147 (64.2) 6 (2.6) 14 (6.1)	49 (62.8) 46 (59.0) 1 (1.3) 2 (2.6)	31 (83.8) 26 (70.3) 0 (0.0) 5 (13.5)	0.107 0.188 0.437 0.005
Rhythm Control Medication on admission Amiodarone Propafenone Sotalol	3 (21.4) 3 (21.4) 0 (0.0) 0 (0.0)	49 (19.8) 32 (12.9) 16 (6.5) 1 (0.4)	80 (18.0) 45 (10.1) 31 (7.0) 4 (0.9)	32 (14.0) 22 (9.6) 8 (3.5) 2 (0.9)	12 (15.4) 8 (10.3) 3 (3.8) 1 (1.3)	4 (10.8) 3 (8.1) 1 (2.7) 0 (0.0)	0.489 0.574 0.450 0.784
ACEi-ARB at discharge	8 (44.4)	110 (41.2)	203 (44.0)	109 (45.4)	40 (46.0)	15 (37.5)	0.877
Statin at discharge	9 (50.0)	85 (31.8)	197 (42.7)	101 (42.1)	38 (43.7)	13 (32.5)	0.043
Clinical Data							
Systolic BP (mmHg)	138.6 (17.1)	136.6 (27.0)	140 (31.0)	143.9 (25.3)	151.9 (26.0)	140.4 (22.9)	<0.001
Diastolic BP (mmHg)	83.5 (11.8)	78 (22.0)	80 (19.0)	80 (20.0)	83.4 (16.7)	85 (23.0)	0.006
NT-pro BNP (pg/mL)	826 (17,307)	468 (2087)	187 (2056)	519 (1717)	152 (1788)	217 (1635)	0.438
High Sensitivity Troponin (pg/mL)	47 (73.0)	25 (38.0)	27 (40.0)	26 (38.0)	28 (41.0)	28 (40.0)	0.803
ECG on admission Sinus Rhythm AF Atrial Flutter	5 (35.7) 7 (50.0) 2 (14.3)	45 (21.1) 148 (69.5) 7 (3.3)	78 (20.9) 253 (67.6) 21 (5.6)	38 (19.9) 139 (72.8) 5 (2.6)	7 (10.9) 53 (82.8) 1 (1.6)	2 (6.5) 26 (83.9) 2 (6.5)	0.079 0.029 0.121
GFR by CKD-EPI (mL/min/1.73 m^2^)	54.6 (30.6)	59.9 (22.2)	61.9 (36.0)	62.6 (36.0)	58.9 (23.8)	64.5 (25.8)	0.267
TTE on admission Left Atrium Diameter (cm) LVEF	5.2 (0.7) 50 (15.0)	4.5 (0.9) 50 (17.0)	4.3 (0.9) 55 (15.5)	4.3 (0.7) 55 (15.0)	4.5 (0.9) 55 (12.9)	5.5 (0.7) 50 (14.0)	0.148 0.071
Hospitalization							
Length of hospitalization (days)	8.7 (5.1)	6 (7.0)	6 (6.0)	6 (6.0)	6 (7.0)	4.5 (6.0)	0.128
Reason for hospitalization AF ACS HF HVD Other	6 (33.3) 4 (22.2) 5 (27.8) 1 (5.6) 2 (11.1)	87 (34.9) 22 (8.8) 79 (31.7) 16 (6.4) 45 (18.1)	175 (41.5) 51 (12.1) 103 (24.4) 21 (5.0) 72 (17.1)	93 (42.7) 22 (10.1) 57 (26.1) 9 (4.1) 37 (17.0)	20 (26.7) 5 (6.7) 30 (40.0) 5 (6.7) 15 (20.0)	16 (43.2) 3 (8.1) 13 (35.1) 0 (0.0) 5 (13.5)	0.096 0.331 0.051 0.554 0.954
Scores							
CHA_2_DS_2_-VASc	4.2 (1.2)	4 (3.0)	4 (3.0)	4 (3.0)	5 (3.0)	4 (3.0)	0.668
HAS-BLED	2 (2.0)	2 (1.0)	2 (1.0)	2 (1.0)	2 (1.0)	1 (1.0)	0.084

Continuous variables: mean (±SD) (for variables with normal distribution), median (IQR = Q3–Q1) (for variables with non-normal distribution), and categorical variables: number (%). The statistically significant results (*p*-values < 0.05) are in bold. ACEi-ARB = angiotensin-converting enzyme inhibitor–angiotensin receptor blocker; ACS = acute coronary syndrome; AF = atrial fibrillation; AMI = acute myocardial infarction; BMI = body mass index; BP = blood pressure; CABG = coronary artery bypass grafting; CAD = coronary artery disease; CHA_2_DS_2_-VASc = congestive heart failure or left ventricular dysfunction, hypertension, age ≥75 (doubled), diabetes, stroke (doubled)–vascular disease, age 65–74, sex category; CKD = chronic kidney disease; CKD-EPI = chronic kidney disease–epidemiology collaboration; COPD = chronic obstructive pulmonary disease; ECG = electrocardiogram; GFR = glomerular filtration rate; HAS-BLED = hypertension, abnormal renal/liver function, stroke–bleeding history or predisposition, labile international normalized ratio, elderly, drugs/alcohol concomitantly; HF = heart failure; HFmrEF = heart failure with mid-range ejection fraction; HFpEF = heart failure with preserved ejection fraction; HFrEF = heart failure with reduced ejection fraction; HVD = heart valve disease; ICD = implantable cardioverter defibrillator; IQR = interquartile range; LVEF = left ventricular ejection fraction; NOAC = non-vitamin K antagonist oral anticoagulants; NT-pro BNP = N-terminal pro-B-type natriuretic peptide; OAC = oral anticoagulant drugs; PCI = percutaneous coronary intervention; SD = standard deviation; TIA = transient ischemic attack; TTE = transthoracic echocardiogram; VKA = vitamin K antagonists.

## Data Availability

The data used for the present study can be requested from the corresponding author (George Giannakoulas, e-mail: g.giannakoulas@gmail.com).
